# Investigating the fluctuating nature of post-COVID pain symptoms in previously hospitalized COVID-19 survivors: the LONG-COVID-EXP multicenter study

**DOI:** 10.1097/PR9.0000000000001153

**Published:** 2024-04-17

**Authors:** César Fernández-de-las-Peñas, Oscar J. Pellicer-Valero, José D. Martín-Guerrero, Valentín Hernández-Barrera, Lars Arendt-Nielsen

**Affiliations:** a Department of Physical Therapy, Occupational Therapy, Physical Medicine and Rehabilitation, Universidad Rey Juan Carlos (URJC), Madrid, Spain; b Center for Neuroplasticity and Pain (CNAP), SMI, Department of Health Science and Technology, Faculty of Medicine, Aalborg University, Aalborg, Denmark; c Image Processing Laboratory (IPL), Universitat de València, Parc Científic, Paterna, València, Spain; d Intelligent Data Analysis Laboratory, Department of Electronic Engineering, ETSE (Engineering School), Universitat de València (UV), Burjassot, Valencia, Spain; e Valencian Graduate School and Research Network of Artificial Intelligence (ValgrAI), València, Spain; f Department of Public Health, Universidad Rey Juan Carlos (URJC), Madrid, Spain; g Department of Gastroenterology & Hepatology, Mech-Sense, Clinical Institute, Aalborg University Hospital, Aalborg, Denmark; h Steno Diabetes Center North Denmark, Clinical Institute, Aalborg University Hospital, Aalborg, Denmark

**Keywords:** COVID-19, Musculoskeletal pain, Post-COVID, Risk factors, Prevalence

## Abstract

**Objective:**

This cohort study used Sankey plots and exponential bar plots for visualizing the fluctuating nature and trajectory of post-COVID pain in previously hospitalized COVID-19 survivors.

**Methods:**

A cohort of 1266 subjects hospitalised because of COVID-19 during the first wave of the pandemic were scheduled for a telephone interview at 8.4 (T1), 13.2 (T2), and 18.3 (T3) months in average after hospitalization for collecting data about post-COVID pain. Patients were asked for about pain symptomatology that was attributed to the infection. Hospitalization and clinical data were collected from medical records.

**Results:**

The prevalence of myalgia as COVID-19–associated symptom was 29.82% (n = 389) at hospitalization (T0). The prevalence of post-COVID pain was 41.07% (n = 520) at T1, 34.29% (n = 434) at T2, and 28.47% (n = 360) at T3. The recovery exponential curve revealed a decrease trend visualizing that post-COVID pain improved over the time span investigated. Pain in the lower extremity and widespread pain were the most prevalent locations. Female sex (OR 1.507, 95% CI 1.047–2.169), pre-existing pain symptoms (OR 1.724, 95% CI 1.237–2.403), headache as onset-symptom (OR 2.374, 95% CI 1.550–3.639), days at hospital (OR 1.012, 95% CI 1.000–1.025), and presence of post-COVID pain at T1 (OR 13.243, 95% CI 9.428–18.601) were associated with post-COVID pain at T2. Only the presence of post-COVID pain at T1 (OR 5.383, 95% CI 3.896–7.439) was associated with post-COVID pain at T3.

**Conclusion:**

Current results show a fluctuating evolution with a decreasing tendency of post-COVID pain during the first years after hospitalization. The development of post-COVID pain soon after SARS-CoV-2 infection predispose for long-lasting chronic pain.

## Abstract 2

Post-COVID pain symptoms exhibit a fluctuating evolution with a decreasing tendency after hospitalization. The development of post-COVID pain soon after SARS-CoV-2 infection predispose for chronic pain.

## 1. Introduction

From the beginning of the coronavirus disease, 2019 (COVID-19) pandemic, it has been observed that symptoms can appear or be persistent once the acute infection has been surpassed. More than 100 long-lasting symptoms affecting multiple systems, eg, cardiovascular, neurological, respiratory, or musculoskeletal, have been described.^14^ Thus, the presence of long-lasting symptoms has received different terms according to the cut-off time frame considered after the acute infection, being long-COVID or post-COVID-19 condition the most commonly used today. The term long-COVID has been proposed for describing the presence of any post-COVID symptom after surpassing an acute severe acute respiratory syndrome coronavirus-2 (SARS-CoV-2) infection.^4^ The term post-COVID-19 condition is defined as a condition “occurring in people with a history of probable or confirmed SARS-CoV-2 infection, usually 3 months from the onset of COVID-19 with symptoms that last for at least 2 months after and cannot be explained by an alternative medical diagnosis.”^31^ In the last years, different meta-analyses have found that up to 50% of COVID-19 survivors suffer from a plethora of post-COVID symptoms lasting from months^2,11^ up to 1^12,13^ or 2^26^ years.

Pain, particular headache and musculoskeletal, is a symptom experienced at both the acute phase of the infection and after the infection in 15% to 20% of subjects who had been surpassed a SARS-CoV-2 infection.^8^ This meta-analysis reported a pooled prevalence rates of post-COVID myalgia, joint pain, and chest pain ranging from 5.65% to 18.15%, 4.6% to 12.1%, and 7.8% to 23.6% during the first 6 months after the infection.^8^ A recent meta-analysis estimated prevalence rates of 8%, 18%, 17%, and 12% for chest pain, joint or muscle-related pain, general body pain, or headache, respectively, 1 year after COVID-19.^15^ Both meta-analyses revealed considerable heterogeneity across pooled data because studies included follow-ups ranging from 1 to 12 months after the infection, samples ranging from 70 to 1,000 patients, and mixed hospitalized and nonhospitalized cohort.^8,15^ In addition, it is notable to remark that studies investigating post-COVID pain but within an overall post-COVID-19 condition have reported lower prevalence rates (10%-20%) than those studies specifically investigating just post-COVID pain (40%-60%).^1,6,17,30,36^

It should be also considered that most studies investigating post-COVID pain symptoms have used cross-sectional designs because they assessed the presence of pain just at one follow-up period.^1,6,8,15,17,30,36^ Understanding the long-term evolution of post-COVID pain symptomatology may have implications for optimizing patient treatment care, public health outcomes, and for informing patients. In a previous study, we analyzed the trajectory of post-COVID pain symptomatology from the onset of the infection up to the first 12 months after hospital discharge in a cohort of individuals who were hospitalized because of SARS-CoV-2 infection.^5^ The present trajectory study is a follow-up including data from the onset of infection up to 18 months after hospital discharge. Two novel methods were applied, the Sankey plots and exponential bar plots, to further visualize of the trajectory of post-COVID pain symptoms.

## 2. Methods

The LONG-COVID-EXP program includes a multicenter cohort study of subjects who had been hospitalized by an acute SARS-CoV-2 infection during the first wave of the pandemic (March to May 2020) in 5 public urban hospitals of Madrid (Spain). Only patients with a diagnosis confirmed by real-time reverse transcription polymerase chain reaction (RT-PCR) assay of nasopharyngeal/oral swab samples at hospital admission were included. As previously described, patients hospitalized in these hospitals (n = 7,150) during the first wave of the pandemic were included in an anonymous database, and a randomly selected sample of 400 subjects from each hospital was invited to participate.^5^ The Ethics Committee of all involved hospitals approved the study (HUFA20/126, HUF/EC 1517, HUIL/092-20, HCSC20/495E, and HSO25112020). All participants provided informed consent before their inclusion and before collecting any data.

### 2.1. Collection data

The procedures of this multicenter cohort study have been previously described.^5^ First, clinical and hospitalization data were collected from hospital medical records. Participants were scheduled for a telephone semi-structured interview by trained health care researchers at 6 (T1), 12 (T2), and 18 (T3) months after hospitalization. A specific questionnaire focusing on post-COVID pain symptoms was developed. We used the definition of chronic primary musculoskeletal pain proposed by the International Association for the Study of Pain for defining musculoskeletal post-COVID pain.^25^ Thus, participants were asked for the presence of pain that appeared after the infection, pain for at least 3 consecutive months, and whether pain persisted at each time of assessment. They were asked to describe the pain location (eg, cervical spine, shoulder, thorax, lower extremity, upper extremity, or generalized) at each follow-up. We excluded headaches because of their particular classification and need for a diagnosis conducted by a neurologist. Participants were excluded if presented any underlying medical condition that can explain their pain, eg, arthritis.

### 2.2. Sankey plots

Sankey plots are visual diagrams used for visualization of the flow of quantitative data permitting the analysis of the fluctuating evolution of a cohort of individuals over time.^24^ The *x*-axis represents each follow-up (6, 12, or 18 months after). The *y*-axis represents the percentage of individuals experiencing (or not) a particular symptom (post-COVID pain). The arcs depict the flows of subjects between the states (positive or negative), with a width that is proportional to the percentage (from the total cohort) of subjects in that flow. The percentage of subjects with or without the symptom is annotated into the right side of the vertical bar, whereas the flows themselves with the percentage of individuals that they contain are annotated into the left side of the vertical bar.^24^

### 2.3. Exponential bar plots

Exponential bar plots were the method for visualization of the trajectory of the symptoms and were created with Matplotlib 3.3.4. The curve slope was fitted according to the following formula $y=Ke^{ct}$, where y represents the modeled prevalence of the symptom (brain fog, memory loss, and concentration loss) at a time t (in months), and K and c are the parameters of the model.^5^

### 2.4. Statistical analysis

Finally, multivariate logistic regressions were performed to identify those variables collected at hospital admission and the presence of post-COVID pain at the first follow-up (T1) associated with the development of post-COVID pain at 12 (T2) and 18 (T3) months after by using Python's library statsmodels 0.11.1. Adjusted odds ratio (OR) with their respective confidence intervals (95% CI) were calculated. A priori, the level of significance was set at 0.05.

## 3. Results

From an initial sample of 2000 previously hospitalized COVID-19 survivors, a final sample of 1,266 patients (45.6% women, age: 61 years, SD: 16 years) participated in all follow-up periods: T1 (mean: 8.4, range 6–10), T2 (mean: 13.2, range 11–15), and T3 (mean: 18.3, range 16–21) months after hospital discharge. Table 1 summarizes COVID-19 associated symptoms at hospital admission, medical comorbidities, and preexisting pain conditions of the final sample.

**Table 1:** Demographic, clinical, and hospital admission data of the sample (n = 1,266).

Age, mean (SD), y	61 (16.5)
Female (%)	578 (45.6%)
Weight, mean (SD), kg	74.5 (14.5)
COVID-19–associated symptoms at hospital admission, n (%)	
Fever	948 (74.9%)
Dyspnoea	361 (28.5%)
Myalgia	374 (29.5%)
Cough	360 (28.4%)
Headache	135 (16.7%)
Diarrhoea	105 (8.3%)
Anosmia	105 (8.3%)
Ageusia	66 (7.0%)
Throat pain	66 (5.2%)
Vomiting	39 (3.0%)
Number of COVID-19–associated onset symptoms, mean (SD)	2.2 (0.8)
Previous medical comorbidities, n (%)	
Hypertension	336 (26.5%)
Diabetes	158 (12.5%)
Cardiovascular disease	141 (11.2%)
Asthma	85 (6.7%)
Obesity	57 (4.5%)
Chronic obstructive pulmonary disease	47 (3.7%)
Rheumatological disease	16 (1.3%)
Number of previous medical comorbidities, mean (SD)	1.0 (0.8)
Previous pain symptomatology, n (%)	517 (40.8)
Previous diagnoses of pain condition, n (%)	
Tension-type headaches	70 (5.5%)
Migraine	46 (3.6%)
Arthritis	34 (2.7%)
Osteoarthritis	186 (14.6%)
Fibromyalgia syndrome	20 (1.6%)
Stay at the hospital, mean (SD), d	10.5 (10.8)
Intensive care unit admission	78 (6.2%)

### 3.1. Fluctuating nature of post-COVID pain symptoms

**Figure 1.**
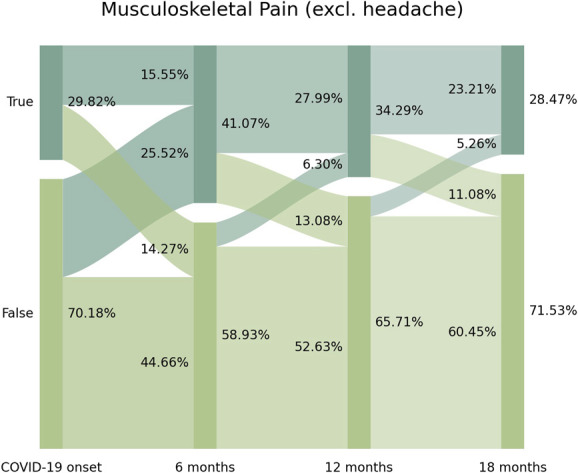
Sankey plots of musculoskeletal post-COVID pain (from left to right) at T0 (hospital admission), T1 (8.4 months after hospitalization), T2 (13.2 months after), and T3 (18.3 months after).

Figure 1 graphs Sankey plots visualizing the prevalence of pain symptomatology. The prevalence of pain as COVID-19 onset-symptom was 29.82% (n = 389) at hospital admission (T0). From this cohort of patients presenting myalgia as an onset symptom, 46.50% (n = 181/389) did not report the symptom after infection (14.27% arc from true at T0 to false at T1), suggesting that pain is an onset symptom that did not persist in almost 50% of patients. The prevalence of post-COVID pain was 41.07% (n = 520) at T1, 34.29% (n = 434) at T2, and 28.47% (n = 360) at T3. Figure 1 revealed a fluctuating pattern of pain symptomatology where 62.10% (n = 323/520) of those reporting pain at T1 developed this symptom “de novo” because they did not reported pain as COVID-19 onset symptom at hospital admission (25.52% arc from false at T0 to true at T1). Similarly, 18.40% (n = 80/434) of patients experiencing pain symptoms at T2 did not report this symptom at T1 (6.30% arc from false at T1 to true at T2), suggesting that a group of patients have a “delayed-onset” in their pain symptoms. Overall, the Sankey plots revealed that 197 patients (15.50% of the sample) reported pain symptoms from the onset of the infection and persisted during all the follow-up periods.

**Figure 2.**
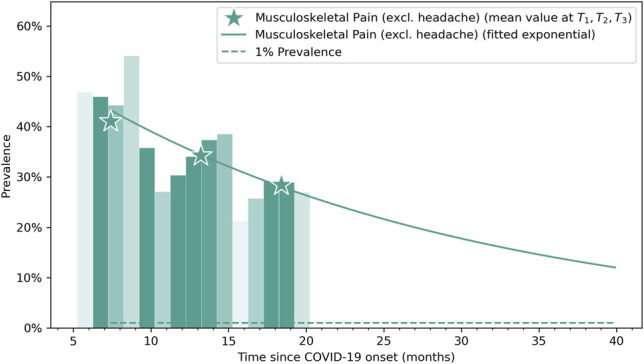
Exponential recovery curve of musculoskeletal pain. The vertical bars represent the percentage of patients that have musculoskeletal post-COVID pain at any time (opacity approximately indicates the sample size at a given time). Asterisks represent mean values at T0, T1, T2, and T3 follow-ups.

The fitted exponential curves visualize a decreased prevalence trend in post-COVID pain symptoms during the following years after the infection (Fig. 2). Vertical bars represent the percentage of patients self-reporting pain at each time-point. The time-point prevalence values at each post-COVID follow-up (T1, T2, and T3) are marked with asterisks in the graph.

**Figure 3.**
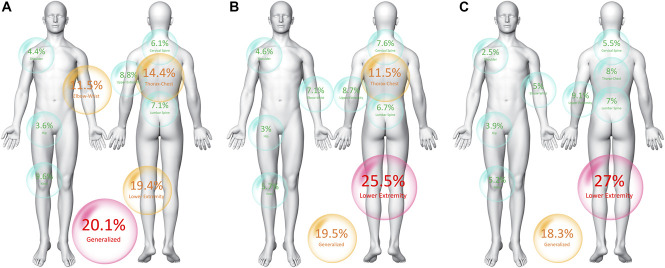
Location of musculoskeletal post-COVID pain symptoms at T1 (A), T2 (B), and T3 (C).

The main body locations of post-COVID pain symptoms at each follow-up period (T1, T2, and T3) can be observed in Figure 3. As it can be observed, pain in the lower extremity and generalized pain symptoms were the most prevalent locations self-reported by the patients.

### 3.2. New-onset post-COVID pain

The prevalence of preexisting pain symptoms before SARS-CoV-2 acute infection was 40.80% in the sample (n = 517). From 520 individuals reporting post-COVID pain at T1, 284 patients (54.60%) reported pain before infection, whereas the remaining 236 (45.40%) started with new pain symptomatology. Thus, from those 284 patients with previous pain symptoms, 129 (45.1%) reported that post-COVID pain was different from their previous symptoms, whereas the remaining 155 individuals (54.9’%) experienced an increase/worsening of their previous pain symptoms (exacerbated post-COVID–related pain). Accordingly, the prevalence of new-onset post-COVID pain symptoms at T1 was 70% (n = 365/517). From 434 patients with post-COVID pain at T2, 252 (58.06%) reported pain symptomatology before infection, where 101 (40.10%) described it as a new (post-COVID) symptom. Thus, the prevalence of new-onset post-COVID pain at T2 was 65.20% (n = 283/434). Finally, from 360 individuals with post-COVID pain at T3, 219 (60.08%) reported pain symptoms before infection. From these 219 patients, just 95 (43.40%) described it as a new symptom. Therefore, the prevalence of new-onset post-COVID at T3 was 65.50% (n = 236/360).

### 3.3. Risk factors associated with long-term post-COVID pain

The multivariate analysis revealed that, after adjusting by all variables, female sex (OR 1.507, 95% CI 1.047–2.169, *P* = 0.03), preexisting musculoskeletal pain (OR 1.724, 95% CI 1.237–2.403, *P* < 0.001), headache as COVID-19–associated symptom at hospital admission (OR 2.374, 95% CI 1.550–3.639, *P* < 0.001), days at hospital (OR 1.012, 95% CI 1.000–1.025, *P* = 0.048) and the presence of post-COVID pain at T1 (OR 13.243, 95% CI 9.428–18.601, *P* < 0.001) were significantly associated with post-COVID pain symptoms at T2. Thus, only the presence of post-COVID pain at T1 (OR 5.383, 95% CI 3.896–7.439, *P* < 0.001) was significantly associated with post-COVID pain at T3 (Table 2).

**Table 2:** Adjusted odd ratio (95% confidence interval) of the multivariate regression analyses of post-COVID pain symptoms at T2 and T3 follow-up periods.

	T2 (13.2 mo)	T3 (18.3 mo)
Age	1.010 (0.993, 1.026)	1.012 (0.999, 1.024)
Female sex	1.507 (1.047, 2.169)*	1.324 (0.934, 1.877)
Weight	0.999 (0.987, 1.012)	1.001 (0.989, 1.013)
Medical comorbidities		
Hypertension	1.040 (0.707, 1.530)	1.237 (0.864, 1.770)
Diabetes	1.455 (0.896, 2.363)	1.139 (0.728, 1.784)
Cardiovascular diseases	0.885 (0.524, 1.494)	1.268 (0.792, 2.030)
Asthma	0.978 (0.527, 1.815)	1.253 (0.710, 2.209)
Obesity	1.121 (0.500, 2.513)	1.118 (0.531, 2.352)
Chronic obstructive pulmonary disease	1.152 (0.494, 2.685)	1.243 (0.563, 2.745)
Rheumatological diseases	3.069 (0.697, 5.515)	3.394 (0.969, 7.886)
Previous musculoskeletal pain	1.724 (1.237, 2.403)†	1.704 (1.243, 2.335)
Symptoms at hospital admission		
Dyspnoea	1.320 (0.910, 1.913)	1.263 (0.899, 1.793)
Cough	1.181 (0.816, 1.710)	1.124 (0.792, 1.596)
Myalgias	1.280 (0.895, 1.831)	1.348 (0.963, 1.887)
Headache	2.374 (1.550, 3.639)†	1.452 (0.973, 2.168)
Diarrhoea	1.155 (0.689, 1.937)	1.243 (0.764, 2.021)
Anosmia	1.331 (0.693, 2.557)	1.253 (0.682, 2.301)
Ageusia	1.385 (0.717, 2.675)	1.134 (0.609, 2.112)
Throat pain	0.730 (0.361, 1.475)	0.757 (0.389, 1.474)
Vomiting	0.762 (0.306, 1.898)	1.422 (0.619, 3.270)
Dizziness	1.910 (0.864, 4.222)	1.957 (0.841, 4.554)
Days at the hospital	1.012 (1.000, 1.025)*	1.008 (0.994, 1.021)
Intensive care unit admission	0.830 (0.482, 1.403)	1.385 (0.913, 2.102)
Musculoskeletal pain symptoms at T1	13.243 (9.428, 18.601)†	5.383 (3.896, 7.439)†

* *P* < 0.05.

† *P* < 0.001.

## 4. Discussion

This is the first cohort study using Sankey plots and exponential bar curves, as 2 visualization approaches, for assessing the fluctuating nature of post-COVID pain in people who had been previously hospitalized because of SARS-CoV-2. Thus, exponential bar plots revealed a progressive tendency decrease in the prevalence of post-COVID pain symptoms during the first years after the infection. Female sex, preexisting history of musculoskeletal pain, the presence of headache as onset symptom at hospital admission and suffering from post-COVID pain soon after the infection were factors associated with long-lasting post-COVID pain.

### 4.1. Fluctuating evolution of post-COVID pain symptoms

Previous meta-analyses including cross-sectional studies had reported an overall prevalence of post-COVID pain symptoms ranging from 15% to 20% during the first 6 months after infection.^8,15^ Our cohort study showed a prevalence of post-COVID pain between 25% and 40% during the first 18 months after the acute infection, prevalence rates higher than those rates reported in studies investigating general post-COVID symptoms, where the prevalence of pain symptomatology did not reach 20% the first 6 months after the infection.^8,15^ Nevertheless, our prevalence rates agree with those found in previous studies focusing on post-COVID pain.^1,6,17,30,36^ It is possible that post-COVID pain can be an underestimated if not specifically investigated because it may be not considered as bothersome as other post-COVID symptoms such as fatigue, dyspnea, or brain fog. The LONG-COVID-EXP study is the largest longitudinal study specifically evaluating the prevalence of post-COVID pain symptoms in the same cohort of patients for a follow-up period longer than 1 year. Thus, an important point is that the exponential bar plots visualized that the prevalence of post-COVID pain, although has a potential decreased tendency with the passage of months and years, could last up to 5 years after the infection.

The use of Sankey plots has permitted to visualize a fluctuating nature of post-COVID pain based on the moment when the symptom appears as previously suggested^3^:

(1) New-onset post-COVID pain: subjects experiencing pain after the acute infection and they did not experience this symptom before (25.52% arc from false at COVID-19 onset to true at T1);
(2) Delayed-onset post-COVID pain: individuals reporting post-COVID pain at a longer follow-up period, ie, with a delayed in time, in relation to the acute infection (6.30% from false at T1 to true at T2, or 5.26% from false at T2 to true at T3);
(3) Persistent post-COVID pain: individuals reporting pain from the acute phase of the infection (myalgias) and throughout all follow-up periods (15.55% of the sample).

These 3 potentials nature (ie, new-onset, delayed-onset, and persistent) of post-COVID symptomatology has been previously proposed,^7^ and the use of Sankey plot has permitted to visualize them. By definition, new-onset and persistent post-COVID pain could be easily attributable to SARS-CoV-2 infection if the symptom started no later than 3 months after COVID-19.^31^ Current data support that pain (myalgia) is one of the most common onset symptoms experienced during the acute phase of infection.^32^ Sankey plots revealed that almost 50% of the individuals experiencing myalgia as onset symptom at hospitalization will not experience post-COVID pain. Thus, up to 60% of the subjects reporting post-COVID pain 8 months after the infection did not report pain as an onset symptom at the acute phase. Individuals who experienced myalgia at the time of infection and also reported post-COVID pain 8, 12, and 18 months after (15.55% of the sample, n = 197) developed “persistent” post-COVID pain. These subjects experienced post-COVID pain throughout all follow-ups. We do not know why this group of patients developed symptoms from the beginning of the infection (real persistent symptoms), and it is possible they exhibit particular features that should be monitored carefully in future studies. In fact, the presence of pain as an onset COVID-19–associated symptom and how early the pain begins could provide guidance on the character and prognosis of post-COVID pain in this group of patients.

In relation to the development of new-onset post-COVID pain, it is important to consider the presence of previous pain symptomatology before the infection. This group of patients could exhibit an exacerbation of their previous pain symptoms (exacerbated post-COVID related-pain) or could develop different symptomatology (new-onset post-COVID–related pain). We observed that around 40% of individuals experiencing previous pain before the infection and developing post-COVID pain described their symptoms as different from their previous pain symptoms (new-onset post-COVID pain).

The case of delayed-onset post-COVID pain is more difficult to exclusively attribute to COVID-19 because pain appears several months after the infection (6.30% from false at T1 to true at T2). A potential hypothesis explaining could be that COVID-19 might trigger a delayed long-lasting inflammatory and pronociceptive process with persistent immune activation, leading to a delayed development of pain.^16^ A second hypothesis could be that viral persistence could also unmask or activate underlying comorbidities, leading to an altered nociceptive processing. Third, COVID-19 psychological surrounding factors, eg, posttraumatic stress disorder, depression, anxiety, or sleep disorders, may also be related to the presence of delayed-onset post-COVID pain.^19^ These hypotheses further confirm current assumption that post-COVID symptoms consist of a complex interaction between biological, psychological, and social factors.^29^ Thus, all these factors should be considered for personalized management of post-COVID pain.^9^

### 4.2. Location of post-COVID pain

Yelin et al.^35^ proposed the term “pain-syndromes pattern or cluster” for describing subjects with post-COVID symptoms where pain is the main complaint. In fact, pain can be the isolated post-COVID symptom experienced by a group of patients. Our study revealed that pain symptoms can appear in any area of the body, being the lower extremity and thorax/chest the most prevalent locations. Pain in the lower extremity as the most prevalent in patients with long-COVID has been also reported by Numan.^22^ It is interesting to note that pain in the extremities is also a prevalent symptom during the acute phase of the infection.^28^ It is possible that location of post-COVID pain is related to the location of pain symptoms experienced during the acute phase of the infection, but we did not collect location of pain at hospital admission.

In addition, almost 20% of the sample reported the presence of widespread pain symptomatology, in agreement also with previous reports.^23,34^ It is possible that this group of post-COVID pain patients develop a pattern similar to those with fibromyalgia or myalgic encephalomyelitis/chronic fatigue syndrome (ME/CFS) where the presence of widespread pain and sensitization-associated symptoms would lead to a nociplastic pain phenotype.^10^ Thus, it has been proposed that the clinical overlap between fibromyalgia, ME/CFS, and post-COVID 19 condition would support similar mechanisms between these 3 conditions.^20^ Identification of this subgroup of patients at a higher risk of developing widespread post-COVID pain symptomatology and, potentially, leading to FMS or ME/CFS could be essential for their management. Nevertheless, it should be considered that we only investigated the presence of post-COVID pain and no other post-COVID–associated symptoms, eg, fatigue, sleep problems, or psychological disturbances that are also present in FMS or ME/CFS.

### 4.3. Post-COVID pain–associated risk factors

We identified female sex, previous history of musculoskeletal pain, the presence of headache as onset symptom at hospital admission, and suffering from post-COVID pain soon after the infection (T1) as factors associated with long-lasting post-COVID pain. That female gender is a risk factor for post-COVID symptomatology is clearly supported by former literature.^33^ Thus, the fact that women experience chronic musculoskeletal pain in a greater extent than men^21^ would also lead to a higher risk of developing post-COVID symptoms. Therefore, future management of post-COVID pain should be applied from a sex perspective considering biological and psychological differences.^18^

Previous history of painful conditions was another factor associated with a higher risk of developing post-COVID pain. We found that up to 40% of our sample experienced pain symptoms before SARS-CoV-2 infection. In this group of patients, 2 situations can be present: (1) an exacerbation of previous pain symptomatology or (2) the development of new pain symptomatology. We found that 40% of patients experiencing previous pain symptoms developed post-COVID pain symptoms, and from this subgroup of individuals, 50% to 55% of patients experienced an exacerbation of their previous symptoms, whereas 40% to 45% reported the development of new pain symptoms after the infection. It is possible that SARS-CoV-2 inflammatory mechanisms (ie, cytokine and interleukin storms) can lead to a hyperexcitability of peripheral and central nervous systems throughout different pathways promoting the development of post-COVID pain or, in predisposed individuals, to worsening of preexisting pain symptoms, as it has been reported in the current study.

Finally, the fact that the presence of post-COVID pain at a shorter follow-up was also associated with the presence of post-COVID pain at longer terms would suggest that monitoring of pain symptoms should be carefully considered soon after the infection to avoid chronification of such pain. In fact, the presence of myalgias at hospital admission and how early the pain begins can provide clinical guidance on the prognosis of future post-COVID pain. This is an interesting finding because the presence of myalgia as an onset COVID-19 symptom is associated with good prognosis for hospitalization,^27^ but it has been found to be a risk factor for long-term post-COVID pain. Furthermore, we also observed that headache as COVID-19 onset symptom was associated with long-term post-COVID pain. Accordingly, careful monitoring of painful COVID-19 onset symptoms may help to identify subjects at a risk of developing long-term post-COVID pain symptomatology. Thus, it is possible that early interventions targeting these COVID-19 onset-symptoms could reduce the risk of developing post-COVID pain.

### 4.4. Limitations

Although this is one of the largest studies with the longest follow-up period to date focusing on post-COVID pain symptomatology, it has some limitations. First, we just collect data from hospitalized patients. Specific longitudinal data about post-COVID pain in nonhospitalized patients is lacking. Thus, this cohort of patients was infected during the first wave of the pandemic, accordingly, with the historical SARS-CoV-2 strain. Because hospitalization rates are lower with current SARS-CoV-2 variants, these data should be considered just on patients infected with the historical strain. Second, we did not collect hospitalization variables, eg, COVID-19 severity or hospitalization treatments, which could promote the development of post-COVID pain. Third, data were self-reported collected over telephone, not face-to-face, a procedure with a potential bias in population-based survey studies, but highly used in COVID-19 literature, particularly in large cohort studies like this one. Fourth, we carefully assessed a large cohort of hospitalized COVID-19 survivors during almost 2 years. Although we specifically asked for pain symptoms started no later than 3 months after the infection, a small proportion of individuals reported post-COVID pain symptoms at T2 and not at T1. Thus, the development of post-COVID pain months after the infection is difficult to exclusively attribute to SARS-CoV-2 as it has been previous discussed. Fifth, because of the large sample size, we did not analyze the phenotype of post-COVID pain symptoms; accordingly, we do not currently know the nature, eg, musculoskeletal or neuropathic, of these pain symptoms. Evidence suggests (based on studies with small sample sizes) that post-COVID pain can present any phenotype, ie, nociceptive, neuropathic, or nociplastic, as described by the IASP.^10^ Finally, it is important to consider that this study just investigated the presence of post-COVID pain and no other post-COVID symptomatology such as fatigue, dyspnea, or brain fog.

## 5. Conclusions

The use of Sankey plots revealed a fluctuating nature of post-COVID pain in people who had been previously hospitalized because of SARS-CoV-2. Thus, exponential bar plots visualized a progressive tendency decrease in the prevalence of post-COVID pain during the first years after an acute SARS-CoV-2 infection. Female sex, preexisting history of musculoskeletal pain, the presence of headache as onset symptom at hospital admission, and suffering from post-COVID pain soon after the infection (T1) were factors associated with long-lasting post-COVID pain. Up to 28% of hospitalized COVID-19 survivors report post-COVID pain after in average 1.5 years.

## Disclosures

The authors have no conflict of interest to declare.
